# Diamond-Like-Carbon Coated Dies for Electromagnetic Embossing

**DOI:** 10.3390/ma13214939

**Published:** 2020-11-03

**Authors:** Marius Herrmann, Björn Beckschwarte, Henning Hasselbruch, Julian Heidhoff, Christian Schenck, Oltmann Riemer, Andreas Mehner, Bernd Kuhfuss

**Affiliations:** 1 Bremen Institute for Mechanical Engineering–Bime, University Bremen, 28359 Bremen, Germany; beckschwarte@bime.de (B.B.); schenck@bime.de (C.S.); kuhfuss@bime.de (B.K.); 2 MAPEX Center for Materials and Processing, University Bremen, 28359 Bremen, Germany; 3 University of Bremen, 28359 Bremen, Germany; heidhoff@iwt-bremen.de; 4 Leibniz Institute for Materials Engineering–IWT, 28359 Bremen, Germany; hasselbruch@iwt-bremen.de (H.H.); riemer@iwt.uni-bremen.de (O.R.); mehner@iwt-bremen.de (A.M.)

**Keywords:** impulse forming, adhesion, micro hard milling, hardened tool steel, a-C:H coating

## Abstract

Electromagnetic forming is a high-speed process, which features contactless force transmission. Hence, punching operations can be realized with a one-sided die and without a mechanical punch. As the forces act as body forces in the part near the surface, the process is especially convenient for embossing microstructures on thin sheet metals. Nevertheless, the die design is critical concerning wear like adhesion. Several die materials were tested, like aluminum, copper as well as different steel types. For all die materials adhesion phenomena were observed. To prevent such adhesion an a-C:H-PVD (Physical Vapor Deposition)-coating was applied to steel dies (X153CrMoV12) and tested by embossing aluminum sheets (Al99.5). By this enhancement of the die adhesion was prevented. Furthermore, the die surface was structured with tribology-effective patterns that were generated by micro hard milling. The embossing quality was topographically analyzed with respect to different initial surface states of the sheets. It was identified that thicker sheets facilitate better embossing results. Moreover, the initial sheet surface has a decisive influence on the embossing quality, whereby the characteristic of the topography showed different susceptibility on the initial sheet surface state.

## 1. Introduction

**Figure 1:**
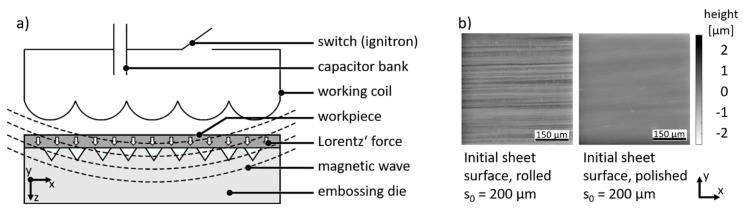
Electromagnetic embossing: (**a**) scheme; (**b**) height profile of initial sheet surfaces.

In electromagnetic forming a contactless force transmission is used to realize a high-speed forming process and applied to processing sheet metal with thicknesses greater than 1 mm [1] as well as tubes [2]. During the process, a high current peak is generated by short-circuiting a capacitor bank via a coil. Thus, an electromagnetic field is generated and an eddy current in an electrically conductive workpiece occurs. Due to the repulsive force (Lorentz’ force) between coil and workpiece, the workpiece is accelerated into an embossing die, see Figure 1. In the case of thin sheets, the penetration depth of the magnetic field is larger than the sheet thickness (skin depth > sheet thickness). Thus, the magnetic wave penetrates the die material as well and hence, an eddy current is generated in the forming die. The impact of such a current was investigated by Cao [3]. He generated the current in the die by using a further tool coil and found that a higher current density in the die enlarges the forming depth. The thickness of the sheet influences also the current density in the primary working coil which in turn affects the magnetic field [4].

Motivated by the advantages of the process like contactless force transmission and thus a one-sided die [5], high strain rates [6] as well as changes in plastic material properties [7], the process is applied to forming [8], cutting [9], joining [10] and embossing [11]. The process is also used to process large parts in way of incremental free forming [12], as well as incremental joining [13]. Besides the macro range the process can also be used in the micro range, so that edge radii down to 120 µm can be achieved without failure during deep drawing of thin sheets [14]. Higher deformation degrees compared to conventional forming are possible and thus the limits of the deformation can be extended [15]. Further advantages in electromagnetic forming are the reduction of wrinkling [16] and springback [17]. Both are especially of great interest for the electromagnetic embossing process. However, the formation of an air cushion can have a negative influence or justify the rebound [18].

Surfaces with embossed microstructures are of growing industrial interest [19], either for optical functions [20] or with tribological effects [21]. The later are used for example on semi-finished sheets for reducing the friction force in dry deep drawing operations [22]. Electromagnetic embossing has been investigated in the macro [23] as well as the micro range [20]. Kamal et al. examined two-stage electromagnetic embossing of microstructures and dealt with problems of significant die wear and sheet-die welding. They concluded that die materials as well as coatings should be investigated to mitigate these problems [11]. First investigations of coated dies have been carried out for electromagnetic forming. DLC (Diamond-Like-Carbon) coatings, soft layers (MoS2) and Ti/TiAlN multilayers on the dies while forming 1.5 mm thick aluminum sheets were investigated and the die wear could be reduced significantly [24]. Vogli et al. suggest DLC coatings to be more suitable due to their better wear resistance [25].

In this study, coated dies for electromagnetic embossing of thin sheets are investigated. For electromagnetic embossing, solutions for reducing the adhesion phenomena needs to be found like die coatings but the coating performance needs to take into account considering eddy current in the die by the penetrating of the magnetic wave. Thus, the adhesion tendency of the workpiece material as well as the die wear are examined. Finally, the embossing of tribology-effective patterns is characterized and evaluated.

## 2. Materials and Methods

### 2.1. Die Manufacturing

At first, different die materials (not coated) were investigated to evaluate the adhesion tendency of aluminum workpiece (Al99.5, EN AW-1050A) to ground die surfaces. Beside aluminum alloy (AlCuMgPb, EN AW-2007), also copper (E-Cu57, EN CW004A) and three steel types were investigated, that is, austenitic chrome-nickel steel (X5CrNi18-10, EN 10088-3) and two cold working die steels (90MnCrV8, EN 90MnCrV8 and X153CrMoV12, EN X153CrMoV12). The X153CrMoV12 was additionally investigated with a polished surface. Only this steel was later used for structuring and coating and additionally an uncoated polished surface, which is typically used as reference surface, was investigated.

**Figure 2:**
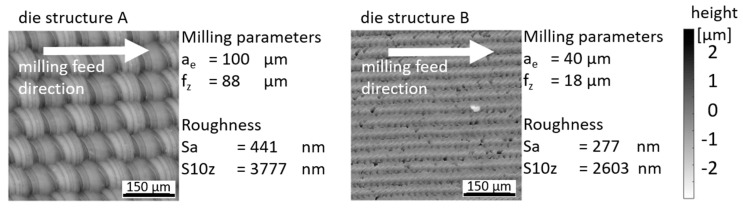
Structure topography with milling parameters and resulting surface roughness values.

In addition to the investigated non-hardened die surfaces, the hardened X153CrMoV12 (62 ± 2 HRC hardness (Rockwell C)) steel was machined by either polishing or by micro hard milling. The polished die was used with an average surface roughness of Sa ~ 6 nm. Micro-structured dies were manufactured using ball-end milling tools with a diameter of d = 2 mm [26]. The cutting process was carried out with a tilted milling tool. Thereby, the pitch angle was 5°. For manufacturing “structure A” the cutting tool was tilted parallel to the feed direction, which lead to a mainly laminar orientated topography. For machining “structure B” the tool was tilted orthogonally to the feed direction, whereby a structure occurred, that is indicated by lines. Further selected milling parameters (feed per tooth f_z_, radial depth of cut a_e_) and the resulting topography with measured roughness values are illustrated in Figure 2. Subsequently, polished and structured dies were sputtered with a DLC-coating.

### 2.2. Diamond-Like-Carbon Coating

**Figure 3:**
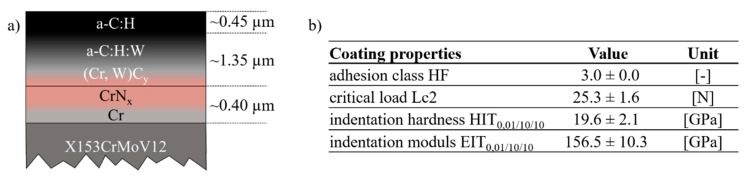
Coating: (**a**) multilayered system; (**b**) primary properties.

An industrial CemeCon magnetron sputtering system CC800/9 SinOx was used for applying the multi-layered coating system on the surfaces of the dies. The coating device was equipped with one chromium, one tungsten and two graphite-targets. The whole PVD process consisted of three phases: the heating phase with a constant heating power of 15 kW for a duration of 1000 s, the substrate plasma etching at a bias voltage of −650 V with a duration of 2500 s at constant gas flow rates of 200 cm^3^/min argon ions and 50 cm^3^/min of krypton ions for activating and cleaning the surfaces and finally the deposition phase starting at a pressure of about 5 mPa. The overall pressure was kept constant at 600 mPa by regulating the argon partial pressure. The krypton gas flow rate during the deposition phase was kept constant at 75 cm^3^/min. The Cr/CrNx layer is acting as a bonding agent for the amorphous layers above, see Figure 3a. The chromium target power was set to 2000 W. The (Cr, W) Cy intermediate layer was graded by continuously decreasing chromium target power and simultaneously activating tungsten target power of 650 W as well as carbon target power of 1500 W. This intermediate layer should further improve both the substrate adhesion and the continuous bonding to the upper functional layers. The functional a-C:H:W layer was deposited by increasing the acetylene (C2H2) gas flow rate up to 35 cm^3^/min and increasing the bias voltage from −50 to −100 V in 1000 s. Hereafter, the PVD parameters were kept constant for a certain time for realizing the layer thickness of about 1.35 µm shown in Figure 3a. The functional a-C:H top layer was applied by grading down and deactivating tungsten target power [27].

The resulting primary coating properties are listed in Figure 3b. The adhesion class is HF 3 measured by six Rockwell adhesion tests [27]. The critical load Lc2 was determined by scratch testing with a Rockwell indenter, which exhibited a tip radius of 0.2 mm [17]. Lc2 represents the first adhesive coating failure by the formation of delamination at about 25 N. A total of 10 scratch tests were carried out for statistical reasons. For determining the indentation hardness HIT0.01/10/10 and modulus EIT0.01/10/10 a total of 49 (7 × 7 array with a lateral measuring field of 2 × 2 mm^2^) micro-hardness indentation tests were performed using a Vickers-diamond. Due to the Bückle law a normal indentation force of 10 mN with a loading, holding and load release duration of 10 s each was set for all single indentations.

### 2.3. Embossing Setup

**Figure 4:**
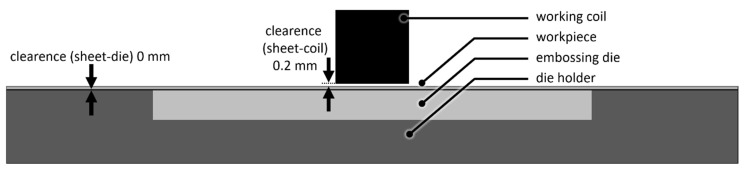
Diagram of the experimental embossing setup.

The used capacitor bank consisted of four parallel connections of a serial capacitor pair (overall eight capacitors) with an overall capacity of C = 100 µF. The maximum voltage of the capacitor bank, which was switched by a single ignitron, is U_max_ = 6 kV. The resulting charge energy of E_max_ = 1800 J was used for all experiments. The coil was a single straight conductor coil made of copper with a rectangular cross-section of 5 × 5 mm^2^. The resulting inductance of the setup was approximately 0.6 µH. The sheet material was Al99.5 with a thickness of s_0_ = 200 µm and 50 µm. The clearance between coil and the 50 × 50 mm^2^ sheet was about 0.2 mm. Between sheet and die was no clearance. The experimental embossing setup is illustrated in Figure 4. The surface of the sheets exhibited grooves caused by rolling as blank manufacturing process, see Figure 1b. The sheets were oriented in the embossing setup, that these grooves were parallel to the single straight conductor coil. Both sheets were processed with no further surface preparation. Additionally, one of the thicker sheet samples was polished. The list of experiments is summed up in Table 1.

In combination with the listed electric setup and the sheet thickness of s_0_ = 200 µm the skin depth ratio (ratio between sheet thickness and skin depth) was 0.301. Finally, to evaluate the die wear the dies were scanned by a digital microscope (Leica DVM6M) (Leica Microsystems GmbH, Wetzlar, Germany) and the amount of adhesion was compared qualitatively in the pictures. The embossing results were evaluated by scans with white light interferometry (Taylor Hobson CCI HD) (Taylor Hobson, Wiesbaden, Germany). In addition to a qualitative visual comparison, a quantitative comparison by Abbott-curves, calculated from the height profiles, was carried out.

## 3. Results

### 3.1. Embossing Die Material

**Figure 5:**
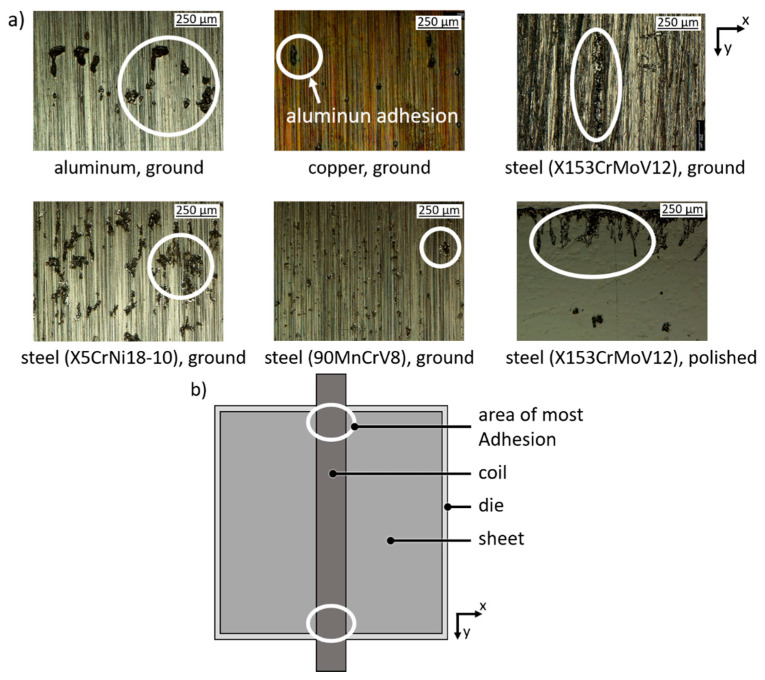
Aluminum adhesion: (**a**) photos of different ground uncoated die materials after embossing; (**b**) sketch of the location with largest identified amount of aluminum adhesion.

All five investigated uncoated die materials were tested in the electromagnetic embossing process. Therefore, aluminum sheets (s_0_ = 200 µm with grooves) were embossed on the dies surfaces. For all die materials, aluminum adhesion (elevated structure during topography scan) could be observed, see Figure 5a. For the polished die of the material X153CrMoV12 more adhesion could be examined compared to the ground surface. Mainly, adhesion occurred directly at the position of the die where the single straight conductor coil was positioned during the process. The largest amount of adhesion could be observed in the area, where the field enhancement effect caused higher Lorentz’ forces. For further investigations of the adhesion on the DLC coating as well as the embossing results the same position was chosen, see Figure 5b.

### 3.2. Diamond-Like-Carbon Coated Dies

**Figure 6:**
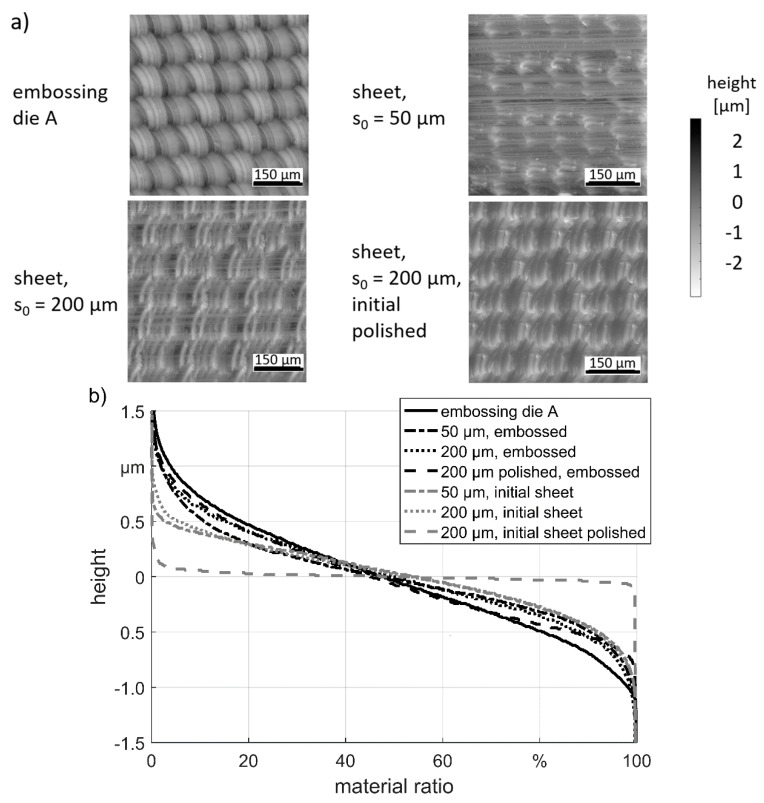
Embossing result “structure A”: (**a**) height profiles; (**b**) Abbott-curves.

After embossing experiments with the different sheets (thickness and initial surface) the surfaces of the three DLC coated dies were investigated by digital microscopy. Especially the edge area which was located under the coil during the process, where the most adhesion at the previous tested dies were observed, was examined. The three DLC coated dies, one polished as reference for the coating as well as both structured, showed no wear. No aluminum adhesion or sheet-die welding could be found. In addition, no coating failure like delamination was detected and thus the suitability of DLC coated dies for electromagnetic embossing of thin sheets is proven. The electromagnetic field penetrates the die with no visible effect on the coating see top left Figure 5a and Figure 6a.

### 3.3. Embossing Result

The height profiles of the dies and the embossed sheets of “structure A” are shown in Figure 6a. After embossing of both sheet thicknesses the sheets exhibit still clearly the groove marks at the surface, compare Figure 1b. Thus, the embossing of the microstructure could not level the initial rolling surface. In the valleys of the rolling grooves, less features were embossed. The embossing result of the sheet with s_0_ = 200 µm was better in comparison with the thinner sheet. This can be explained by the more efficient energy transfer to the thicker sheet and thus a higher force. The previous polishing of the thick sheets led further to an improvement of the embossing result. This can be explained by the removal of the rolled surface by polishing. Also, the cold hardened topography of the rolling which could affect the embossing process is removed by the polishing.

The Abbott-curves of the die with “structure A” as well as of the initial sheets surfaces were compared with the embossing results see Figure 6b. The Abbott-curve of the embossing result was rotated by 180°. The Abbott-curves of the initial polished sheet shows a typical course with a broad plateau. The polished sheet, which showed the best embossing result by visual inspection, fits also best to the Abbott-curve of the die structure. The deviation is biggest in the range of material ratio values between 80–100%, which describes the replication quality of the valleys of the structure. This is the part which is most difficult to emboss. However, the influence of the rolling direction and thus the orientation of the rolling structure must be taken in account. The curve of the embossed sheet with a thickness of s_0_ = 50 µm confirm the visual inspection and exhibit the highest deviation to the Abbott-curve of the die.

**Figure 7:**
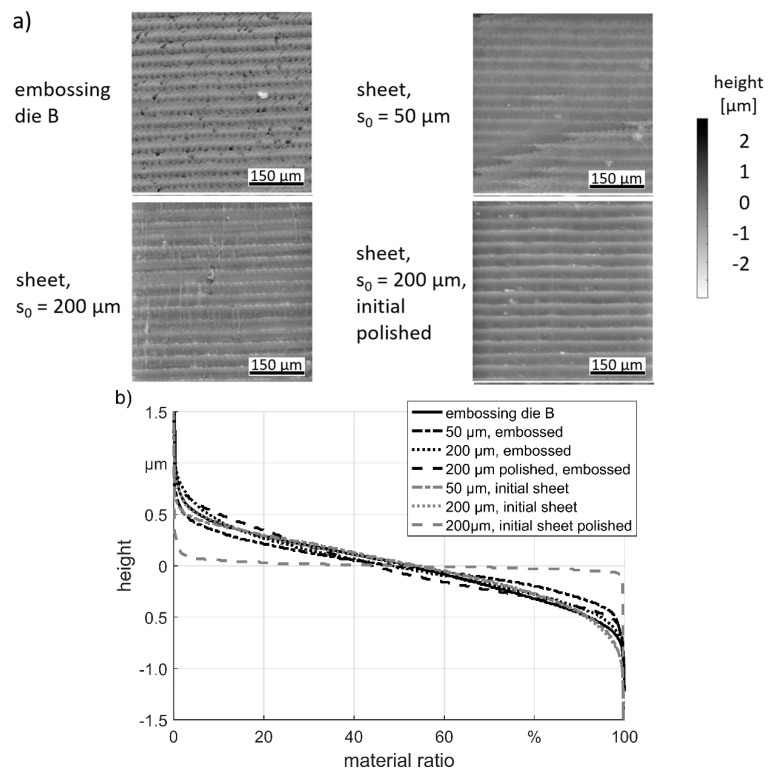
Embossing result “structure B”: (**a**) height profiles; (**b**) Abbott-curves.

The height profiles of “structure B” show fewer differences in the embosoming results see Figure 7. Only at the thick sheet an additionally overlain structure is visible which is orthogonal to the initial rolled structure of the sheet. By comparing the Abbott-curves, it is noticeable that the curves of the rolled initial surfaces are quite similar to the die curve.

The “structure A” showed a stronger influence of the initial surface and the sheet thickness on the embossing result. On the one side “structure A” is higher compared to “structure B” and in the other side the structure characteristic of “structure B” with its line characteristic is more similar with the rolling surface. Thus, the embossing quality depends strongly on the structure, which needs to be embossed.

## 4. Conclusions

In this study, dies for electromagnetic embossing of microstructures in thin aluminum sheets were investigated. Experiments with different die materials as well as coated steel dies were carried out. Two different microstructures on coated dies as well as two different sheet thicknesses were examined. The following conclusion could be drawn:

- Adhesion occurred for all die materials during embossing of aluminum. The highest amount of adhesion was observed under the coil at the edge of the die where the electrical field is assumed highest.
- Diamond-Like-Carbon coating was suitable to prevent the adhesion even during electromagnetic embossing operations of thin sheets when the coating is penetrated by the electromagnetic field.
- No wear of the coating was observed, neither adhesion nor delamination.
- The surface preparation of the sheet with a thickness of s_0_ = 200 µm led to significant differences in the embossing quality of the mainly laminar orientated “structure A”. On polished sheets, this die produced a better embossing quality, whereas on the surface of a rolled sheet still imprints of the rolling marks were found.
- The sheet with a thickness of s_0_ = 50 µm exhibited worse embossing results compared to the thicker sheet with the same initial surface condition, due to less energy transfer.
- For “structure B” which is indicated by line the embossing results showed less differences, thus the initial surface and the chosen process parameter were more suitable for this structure.

Finally, it can be concluded that electromagnetic forming is a promising forming process for thin sheet metal but the initial surface as well as the process needs to be adapted to the embossing die to yield optimal results.

## Figures and Tables

**Table 1:** List of experiments.

No	Al99.5 Sheet (50 × 50 mm^2^)	Embossing Die	Embossing Parameters
1	s_0_ = 50 µm	structure A	overall capacity; C = 100 µF
2	s_0_ = 200 µm		voltage; U_max_ = 6 kV
3	s_0_ = 200 µm, initial polished		charge energy; E_max_ = 1800 J
4	s_0_ = 50 µm	structure B	inductance of the setup, ~0.6 µH
5	s_0_ = 200 µm		coil material, copper
6	s_0_ = 200 µm, initial polished		coil cross-section, 5 × 5 mm^2^
